# Abstract of the Proceedings of Buffalo Medical Association

**Published:** 1871-08

**Authors:** W. C. Phelps

**Affiliations:** Secretary; Buffalo


ART. IV.—*Abstract of the Proceedings of the Buffalo Medical Association.*

---

BUFFALO, August 1st, 1871.

The President, Dr. Johnson, in the Chair.

Dr. Miner reported a case he had recently attended with Dr. Kamerling, of gun-shot wound of the arm and forearm. The patient, a child, received a charge of shot through the left arm and also the forearm, which, at the time, was bent so as to be on the same level. The humerus was shattered so that about three inches of its shaft were removed, and about the same amount from both the ulna and radius. There was no pulsation of the arteries perceptible at the wrist, nor in the arm. The muscular tissues were extensively destroyed, and amputation seemed imperative. As neither the shoulder nor elbow joints were implicated, and having in mind the wonderful results constantly obtained by conservative surgery, they determined to make an attempt to save the arm. The extremities of all the bones were resected, and all the loose pieces of bone removed, and the wounds carefully cleaned; a large part of the ulna was found destroyed. The arm was then laid on a splint and warm water dressing applied. There was at no time any severe hemorrhage. The next day the patient was much improved, had suffered but little pain, and the circulation in the forearm was nearly normal. It is now two weeks since the receipt of the injury, and the progress of the case is, in every respect satisfactory, and there is no doubt of a successful result as to saving the arm, and probably a useful one. It is well known that a wooden or artificial arm is of little benefit to the wearer. If a hand could be saved to a child, it would be useful if only as an ornament, and an arm with no bone in it would be more serviceable than any artificial substitute. The arm in this case will, no doubt, be considerably shortened, but will be very useful. If it can become an established rule in surgery, that an arm with such an injury as this can be saved, we have accomplished what, not long ago, would not have been attempted. During the late war, no doubt many limbs were amputated which might, if proper care could have been bestowed on them, have been saved, and been of immense service to their possessors, especially is this the case with the upper extremities.

Surgically considered, a thumb is of as much use as a leg, and owing to its importance, as much as possible of this small member should be saved.

Dr. M. had taken out one-half of the second phalanx, and had a good and useful thumb, and has even removed the whole of it with fair result.

Another case of injury of the arm was related. A young man was coupling cars, and his arm was caught between them. The soft parts in front of the elbow joint were very badly lacerated,and the ulna fractured, and the brachial artery entirely obliterated at this point, so that there was no pulsation perceptible at either the ulna or radial arteries. There was some circulation noticed on the back of the arm, through the anastomosing vessels. The hand was livid, and symptoms indicated the possible necessity of amputation. This last resort was, however, held in reserve, and an attempt made to save the arm. The next day it had improved much in appearance. The circulation was very good, and now, at the end of a week, the patient was sitting up, feeling quite well, and there are flattering prospects of a serviceable arm.

There is probably great risk of pyæmia in those cases, but not sufficient to counterbalance the resulting good.

Dr. Strong remarked that he was glad that surgeons were paying more attention to conservative surgery than formerly. Amputation should be held in reserve, especially in injuries of the upper extremities. A poor arm is better than no arm, or an artificial one, and even a hand, especially a right hand, is of immense value. In the army, innumerable arms were sacrificed which might have been saved. In a case which he treated with Dr. Miner, some time since, more of the humerus was removed than had been recorded at any previous time; it was a triumph of conservative surgery of itself. The case was complicated by a severe wound across the chest, which laid bare the rib, a portion of which was removed, but in spite of this severe complication, the treatment of the arm was a success. Two-thirds of the upper portion of the humerus was removed, but the patient now has perfect use of the arm below the elbow, and can raise the hand to his head.

Is of the opinion that in almost any injury to the bones of the arm, it may be saved if the circulation is intact. The joints, when destroyed, can be exsected, and a useful arm saved. Saw a case of exsection of the elbow joint in the army, after the battle of Antietam. The case went on two weeks, and it seemed that amputation would have to be performed, but concluded first to exsect the joint. Both ends of the articulating bones were sawn off, and the arm dressed in a semi-flexed position. In one year after the patient could write with the arm. Dr. S., also thinks that pyæmia may be counteracted, and the poison eliminated from the system by the use of quinine. He had much experience with this disease and its treatment in army hospitals, and quinine was his chief remedy.

Dr. Wetmore saw a case similar to that reported by Dr. Miner, in Cleveland last winter, where the circulation in the radial and ulna arteries was destroyed by pressure and contusion of the brachial at the elbow. The circulation was, after a time, fully restored through the anastomotica magna, and the intrasseous arteries. Is of the opinion that quinine is of great service in the treatment of pyæmia, which he believed to be more potable after severe injuries where amputation was not performed, than followed that operation.

Dr. Slocum related a case of dislocation of the humerus downward into the axilla, which he attended last spring. For the first five days after the reduction of the dislocation all progressed well, but on the sixth there was much swelling and pain, especially in the hand. Reduced the swelling as well as possible, and then the parts became edematous. At the seventh week again there was severe inflammation. Counsel was called in and amputation advised. This extreme measure, however, was not carried out. At present there is considerable swelling of the arm. The patient has good use of the elbow joint, and perfect use of the fore-arm and hand. Thinks that the train of unfavorable symptoms following the reduction of the dislocation was due to injury of the brachial nerve at the axilla.

Dr. Miner said that Dr. Strong had intimated in his remarks, that surgeons generally had not faith in quinine for the treatment of pyæmia which it deserves. He thought that physicians might have too great confidence in its power in the treatment and prevention of pyæmia. Support of the system against the depressing effects of the poison circulating in the blood, was of the greatest benefit, and quinine would in a degree assist, but he did not believe that it had any specific anti-poison properties, as claimed by Dr. Strong. When given in one grain doses its effects are not noticeable on the system. In larger doses, it is of value as an antidote to the malarious poisons, and when its good effects were seen in the army, he believed they were due to its control over malarial diseases, and not to any specific action on the pyæmic poison, and the same remark would apply to its use here at the present time.

Dr. Strong replied that quinine, or rather Cincho-quinia, for he favors the active properties of the bark, rather than its alkaloids, produce both tonic and antiseptic effects, and if it will counteract malarial poisons, why will it not also control, by its tonic effects on the system, and counteract, by its specific powers, other blood poisons. He regards it as a specific and tonic, when given in sufficient quantity. It will also vastly restrain suppuration. When in charge of a large army hospital, he used large quantities in all cases of suppurating wounds, and the effect was plainly visible in repressing suppuration and promoting their healing. It should be associated with stimulants and aliments.

Dr. Miner said that as Dr. Strong so readily stepped from the poison of pyæmia to the malarial poison, and claimed quinine as a specific for both, both being blood poisons, he could as well go a little farther, and include the poison of syphilis, diphtheria, scarlatina, &c., all of which, he would not deny, were blood poisons, but quinine, it is well known, is not a remedy of any value as a specific against syphilis or the other diseases named. If it has anything to do in the elimination of blood poisons, it does so by virtue of its tonic properties, and anything that will sustain the system, will assist this process of elimination, but this can not be called a result of any specific power.

Some further discussion at this point followed between these gentlemen, when the association adjourned.

W. C. PHELPS, *Secretary.*

